# 1034. Risk Score for the Need of Mechanical Ventilation in Adults Presenting with Encephalitis.

**DOI:** 10.1093/ofid/ofac492.875

**Published:** 2022-12-15

**Authors:** Ashley N Heck, Denisse Ramirez, Alejandro Granillo Ibanez, Rodrigo Hasbun

**Affiliations:** McGovern Medical School, UTHealth Science Center, Houston, TX, Houston, Texas; McGovern Medical School, UTHealth Science Center, Houston, TX, Houston, Texas; McGovern Medical School, UTHealth Science Center, Houston, TX, Houston, Texas; UT Health Mc Govern Medical School, Houston, Texas

## Abstract

**Background:**

The purpose of this study was to stratify risk subgroups for mechanical ventilation in adults presenting with encephalitis.

**Methods:**

A retrospective observational study of 271 adults ( > 18 years old) admitted with an encephalitis diagnosis according to International Encephalitis Consortium guidelines were enrolled from sixteen hospitals in Houston, Texas between the years of 2005 and 2015.

**Results:**

Mechanical ventilation (MV) was utilized in 91/271 (33.6%) adults with encephalitis and it was associated in hospital mortality (24.2% vs 3.9%, P< 0.001). There were no significant differences in age, race, gender, underlying comorbidities or immunosuppressive conditions, fever, symptoms on presentation, focal neurological deficits, Glasgow coma scale, CSF profile, and abnormal cranial imaging including cerebral edema between patients with or without MV (P >0.05). Risk factors associated with the need for MV included seizures, serum white blood cell (WBC) counts >11,000/mm3, a Sequential Organ Failure Assessment (SOFA) score >3 and abnormal Electroencephalogram (EEG) (P< 0.05). A risk model was created using 3 baseline variables independently associated with MV (P< 0.05): serum WBC count >11,000/mm3, a

SOFA >3 and abnormal EEG. The risk score classified patients into 3 subgroups for the need for MV: low risk [none of the three variables, 1/32 (3.1%)], intermediate risk [1 of the 3 variables, 19/106 (17.9%], and high risk [2 or 3 of the 3 variables, 71/136 (52.2%)].

Risk Classification for Mechanical Ventilation in Patients with Encephalitis.

**Figure:**
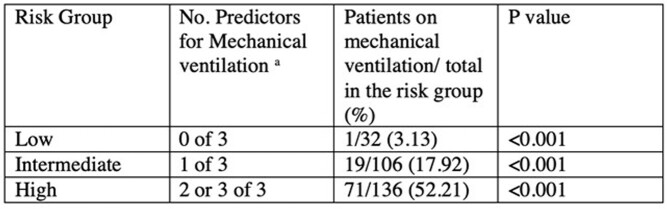

a Predictors include white blood cell count > 11, Sequential Organ Failure Assessment (SOFA) scores > 3 or abnormal Electroencephalogram (EEG)

Receiver Operating Characteristic (ROC) curve of risk score (Low =0 variables seen in the patients, Intermediate = 1 variable seen, and High = 2 or 3 variables seen).

**Figure:**
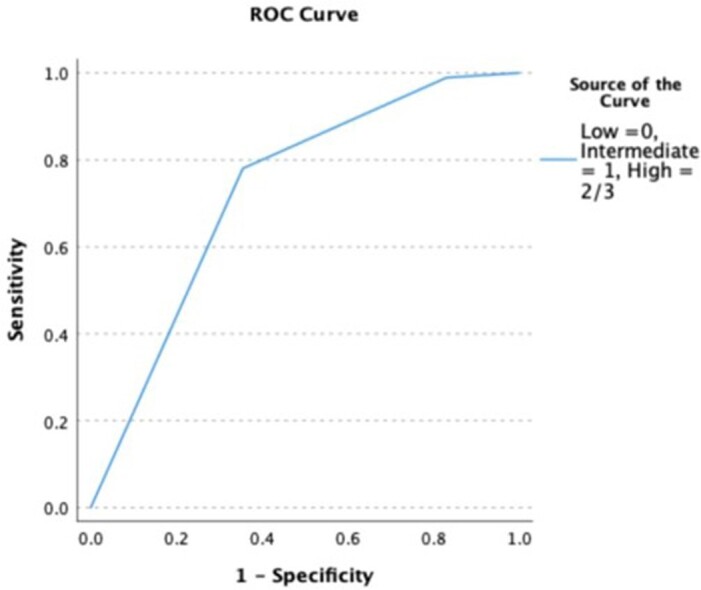

Area under the curve = 0.728.

**Conclusion:**

This risk score can be utilized by clinicians to assess the need for MV in adults presenting with encephalitis to help stratify patients that require intensive care unit admissions.

**Disclosures:**

**Rodrigo Hasbun, MD MPH FIDSA**, Biofire: Grant/Research Support|Biofire: Honoraria.

